# A Phytochemical Approach to Promotion of Self-renewal in Murine Spermatogonial Stem Cell by Using *Sedum Sarmentosum* Extract

**DOI:** 10.1038/s41598-017-11790-0

**Published:** 2017-09-12

**Authors:** Sang-Eun Jung, Yong-Hee Kim, Sunghun Cho, Bang-Jin Kim, Hee-Seok Lee, Seongsoo Hwang, Geun-Bae Kim, Young-Hyun Kim, Myung-Geol Pang, Sanghyun Lee, Buom-Yong Ryu

**Affiliations:** 1 Department of Animal Science & Technology, Chung-Ang University, Anseong, Republic of Korea; 2 Department of Integrative Plant Science, Chung-Ang University, Anseong, Republic of Korea; 3 Department of Cancer Biology, Perelman School of Medicine at the University of Pennsylvania, Philadelphia, PA 19104 USA; 4 Food Safety Risk Assessment Division, National Institute of Food and Drug Safety Evaluation, MFDS Korea, Chungcheongbuk-do Republic of Korea; 5 Animal Biotechnology Division, National Institute of Animal Science, Rural Development Administration, Jeollabuk-do, Republic of Korea; 6 National Primate Research Center, Korea Research Institute of Bioscience and Biotechnology (KRIBB), 30 Yeongudangi-ro, Ochang-eup, Cheongwon-gu, Cheongju-si, Chungcheongbuk-do Republic of Korea; 7 Department of Functional Genomics, KRIBB School of Bioscience, Korea University of Science and Technology (UST), 217 Gajeong-ro, Yuseong-gu, Deajeon Republic of Korea

## Abstract

Spermatogonial stem cells (SSCs) are the basis of spermatogenesis, which is dependent on the ability to self-renew and differentiation. Controlling self-renewal and differentiation of SSCs could apply to treatment of disease such as male infertility. Recently, in the field of stem cell research, it was demonstrated that effective increase in stem cell activity can be achieved by using growth factors derived from plant extracts. In this study, our aim is to investigate components from natural plant to improve the self-renewal of SSCs. To find the components, germ cells were cultured with comprehensive natural plant extracts, and then the more pure fraction, and finally single compound at different concentrations. As a result, we found 5H-purin-6-amine at 1 µg/mL, originated from *Sedum sarmentosum*, was a very effective compound induced SSCs proliferation. Our data showed that germ cells cultured with 5H-purin-6-amine could maintain their stable characteristics. Furthermore, transplantation results demonstrated that 5H-purin-6-amine at 1 µg/mL increased the activity of SSCs, indicating the compound could increase true SSC concentration within germ cells to 1.96-fold. These findings would be contributed to improve further reproductive research and treat male infertility by using natural plant extracts.

## Introduction

Spermatogonial stem cells (SSCs) are the basis of spermatogenesis, which depends on the ability of self-renew, and thus produce large numbers of daughter cells that undergo differentiation^1^. In mice testes, the proportion of SSCs is ~0.01% of total testes cells^2^. Despite the small proportion, SSCs are essential in spermatogenesis process because theoretically 4,096 sperms can be produced from only one stem cell^3^. In addition, SSCs are the only adult stem cells that transmit genetic information from parents to the next generation. Therefore, SSCs could be applied to various research fields related to male reproduction and treatment of male infertility^4^, and by controlling their self-renewal and differentiation characteristics, they could be used to treat disease.

Recently, approaches to using small molecules have become increasingly powerful tools for stem cell biology and reprogramming^5^ because these types of molecules are involved in various mechanisms, such as signaling, metabolism, transcription, and epigenetics^6^. Although small molecular approach is still in the experimental stage, it is currently considered as a promising approach to stem cell research.

Plant extracts are good experimental resources because plants are exposed to severe environmental changes like cold and dry seasons, and synthesize useful molecules to resist these extreme changes^7–10^. Moreover, such plant extracts have been proven to have positive effects on human health^11–14^. Previously, plant-derived drugs were used to treat diseases worldwide. Some studies showed that natural plant extracts can alleviate cancer, metastasis, and inflammation^15, 16^. Other studies also reported the anti-protozoal and anti-inflammatory activity of plant-derived natural products, which are promising candidates for the treatment of contagious diseases^17^. It has been also verified that plant extracts improve sperm motility and function, therefore increasing male fertility^18, 19^.

Moreover, in the field of stem cell research, it was demonstrated that effective stem cell fate regulation can be achieved by controlling the signaling pathway using growth factors derived from plant extracts^20^. Zaret^21^ showed that embryonic stem cells (ESCs) cultured *in vitro* with a plant-derived molecule increased their ability to differentiate. Furthermore, it has been reported that plant extracts enhanced the recovery rate and function of hematopoietic stem cells and injured kidney cells, respectively^22, 23^.

Based on these previous results, we hypothesized that the 11 natural plants, especially related to improvement of sperm motility^24, 25^, would also promote SSC self-renewal and proliferation. Therefore, we selected and used the 11 plant extracts which have potential ability to proliferation of SSC in this experiment. Among many plants, *Sedum sarmentosum* (*S. sarmentosum*) has been frequently used for the treatment of disease in Asian countries^26, 27^. Because the *S. sarmentosum* extract can induce anti-angiogenesis, it might play an important role as an anti-implammatory and anti-nociceptive agent^28^. It has also been indicated that the *S. sarmentosum* alkaloid fraction inhibits the proliferation of murine and human hepatoma cell line^26^. Moreover, Kim *et al*. found that *S. sarmentosum* can be administered to menopausal women due to its estrogenic activities^29^. Thus, *S. sarmentosum* extract might be involved in the regulatory mechanism of various cells.

The aim of this study was to identify a molecule that can maintain self-renewal of SSCs and thus promote cell proliferation. This information may contribute to a new drug database and provide novel insights into male infertility treatment because no studies have investigated the effect of natural plant extract on SSC proliferation until now.

## Results

### Screening the Effect of Plant Extracts on Spermatogonial Stem Cell Proliferation

**Figure 1:**
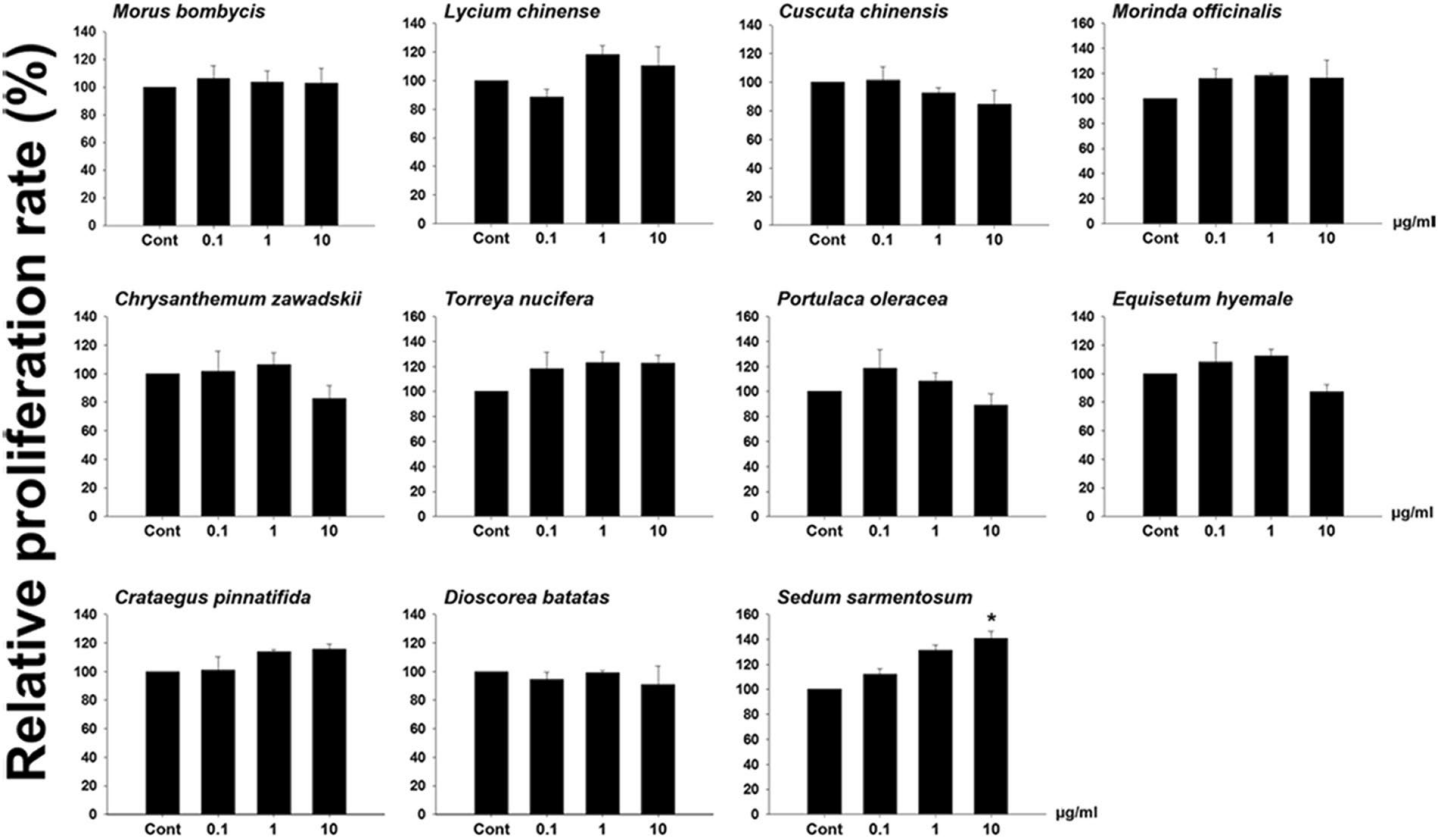
Evaluation of germ cell proliferation cultured with natural plant-derived extracts. Total 11 natural plant derived extract were used in cell culture medium at concentrations of 0.1, 1, or 10 µg/mL to measure the proliferation of cultured germ cells after 1 week of exposure. Values are mean ± SEM (n = 3 established independent cultures for each treatment). Asterisk indicates significant difference (*P* < 0.05) compared to the control.

To evaluate the most effective natural plant extracts, spermatogonial stem cells were cultured for 1 week and then compared cell growth rate between control and treatment groups. Because GDNF is well known as a critical factor for self-renewal of germ cells enriched for SSCs in a serum-free condition, it was added to all treatments and control groups. Germ cells enriched for SSCs proliferation rate was observed with variations due to the effects of various natural plant extracts. The proliferation rate determined slightly increase in a dose-dependent manner, while germ cells cultured with extracts from *Morinda officinalis*, *Torreya nucifera*, and *Crataegus pinnatifida* was not statistically significant. Unlike the above extracts, the effect of *S. sarmentosum* extract at a concentration of 10 μg/mL was significantly different compared with the control group (Fig. 1). Therefore, *S. sarmentosum* extract was selected for fractionation for further experiments because it exerted the greatest effect on germ cell proliferation including SSCs.

### Evaluation of Germ Cell Proliferation Cultured with *Sedum sarmentosum* Fractions

**Figure 2:**
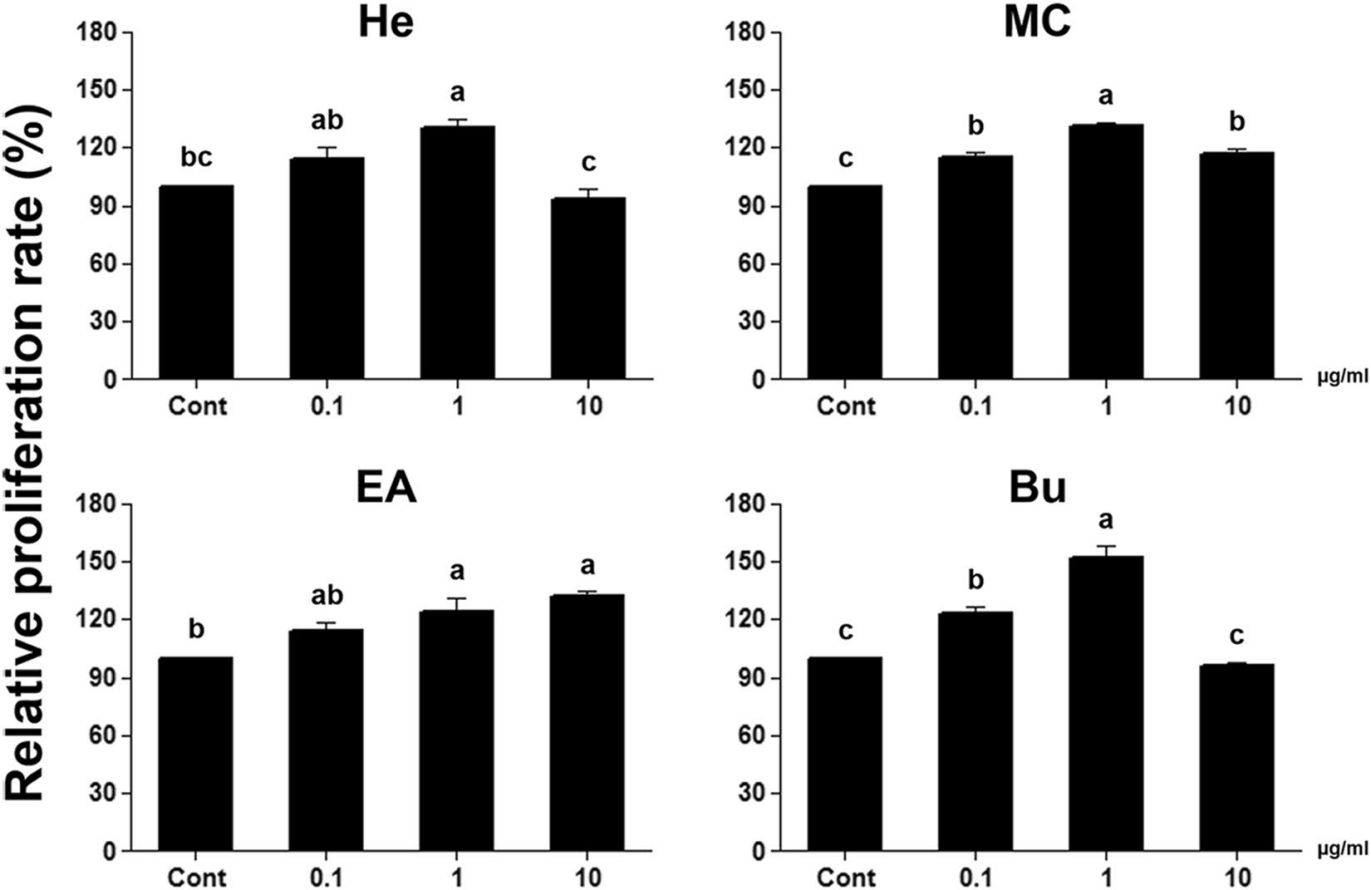
Comparison of germ cell proliferation rates between groups treated with *Sedum sarmentosum* fractions. Relative proliferation rates were evaluated compared to the control by counting the cells after 1 week culture with different *S. sarmentosum* fractions. Proliferation effect on germ cells after culture with four fractions from *S. sarmentosum* at concentrations of 0.1, 1, or 10 µg/mL. Values are mean ± SEM (n = 4). Cont, control; He, *n*-hexane fraction; MC, methylene chloride fraction; EA, ethyl acetate fraction; Bu, *n*-butanol fraction. A statistically significant difference (*P* < 0.05) is displayed as different letters at the top of the column.

The proliferation rate of germ cells was increased in all treatment group compared to the control except for Bu at 10 μg/mL and He at 10 μg/mL. In each treatment groups, the highest proliferation rate was 129.9 ± 4.9%, 131.2 ± 1.9%, 131.9 ± 3.0%, and 151.6 ± 6.6% in EA at 1 μg/mL, MC at 1 μg/mL, EA at 10 μg/mL and Bu at 1 μg/mL, respectively. Among the experimental groups, the highest increase (151.6 ± 6.6%; *P* < 0.05) in the proliferation rate of germ cells occurred in cells cultured with Bu at 1 µg/mL (Fig. 2). Therefore, Bu of *S. sarmentosum* was selected for further investigations.

### Assessment of Compounds from *n*-butanol Fraction of *Sedum sarmentosum* on Germ Cell Proliferation

**Figure 3:**
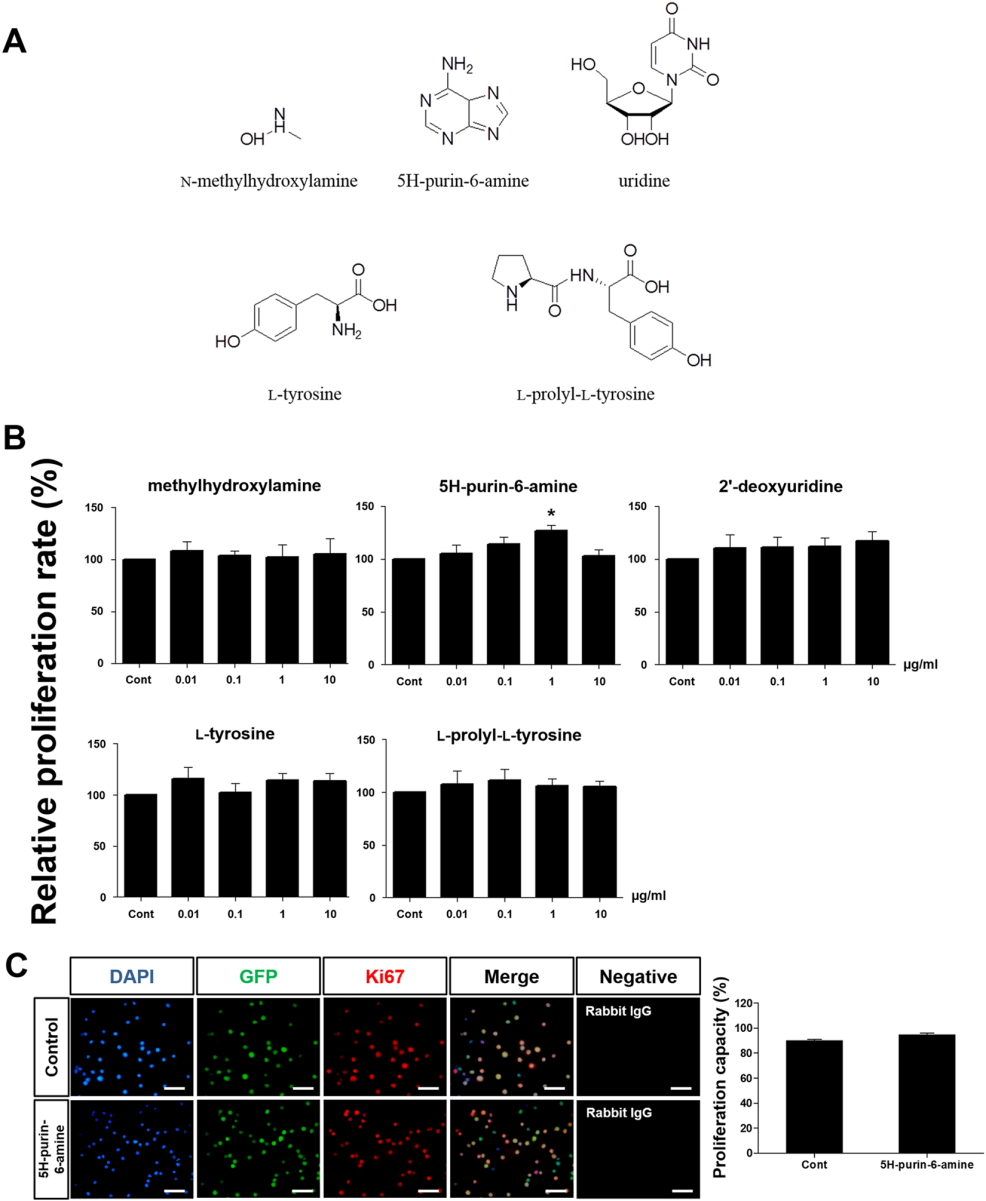
Effect of Sedum sarmentosum compounds on germ cell proliferation. (**A**) Chemical structure of compounds from *n*-butanol fraction of *Sedum sarmentosum*. (**B**) Proliferation effect on germ cells after culture with compounds at concentrations of 0.01, 0.1, 1, or 10 µg/mL. (**C**) Proliferation capacity of germ cells treated by 5H-purin-6-amine 1 μg/mL (Blue, DAPI; Green, GFP; Red, Ki67). Values are mean ± SEM (n = 5). (**B**) n = 5; (**C**) n = 3. A statistically significant difference (*P* < 0.05) is displayed as asterisk at the top of the column. Scale bars; (**C**) = 100 μm.

A portion of the Bu was subjected to MPLC on silica gel eluted with a gradient of CHCl_3_-MeOH to obtain 5 compounds (Bu 2, Bu 6-3, Bu 8-3-3, Bu 9-4-5, and Bu 9-5-5). The chemical structures of Bu 2, Bu 6-3, Bu 8-3-3, Bu 9-4-5, and Bu 9-5-5 were identified as N-methylhydroxylamine, 5H-purin-6-amine, uridine, l-tyrosine, and l-prolyl-l-tyrosine, respectively (Fig. 3A). Germ cells were cultured in a serum-free medium containing each compound at concentrations of 0.01, 0.1, 1, or 10 μg/mL for 1 week. Except for 5H-purin-6-amine, as shown in Fig. 3B, the proliferation rate of germ cells enriched for SSCs was not significantly different from the control for N-methylhydroxylamine, uridine, L**-**tyrosine, and l **-**prolyl-l **-**tyrosine, irrespective of concentration. Although no significant difference was observed in the 5H-purin-6-amine at concentrations of 0.01, 0.1, or 10 μg/mL, a significant increase was observed only for 5H-purin-6-amine 1 µg/mL (127.0 ± 5.9%; *P* < 0.05; Fig. 3B). These results suggested that 5H-purin-6-amine might have proliferation effects on mouse germ cells, including SSCs, *in vitro*. Furthermore, as shown in Fig. 3C, we verified the capacity of germ cells to proliferation by expression of Ki67 as marker for proliferation capacity. The percentage of Ki67 expression was 90.0 $\pm$ 0.9% and 94.5 $\pm$ 1.6% in control and 5H-purin-6-amine 1 μg/mL respectively (Fig. 3C). Because there was no significant difference between control and 5H-purin-6-amine 1 μg/mL as shown in proliferation capacity data, effects of *S. sarmentosum* could be examined by proliferation rate which is the number of germ cells compared with control after 1-week culture.

### Characterization of Germ Cells Cultured with 5H-purin-6-amine Isolated from *Sedum sarmentosum*

**Figure 4:**
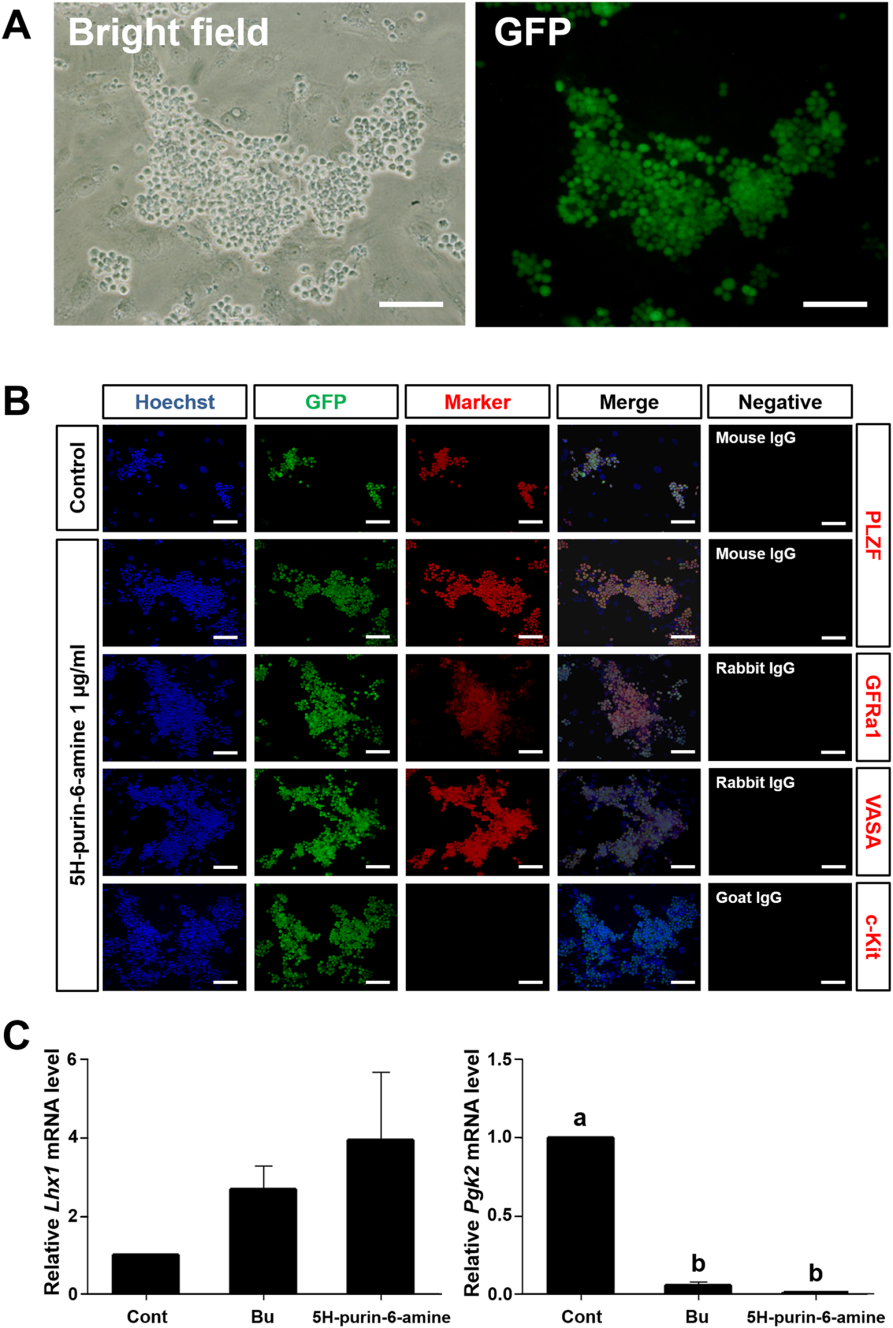
Characterization of germ cells cultured with *n*-butanol fractions or 5H-purin-6-amine. (**A**) EGFP-positive germ cells enriched for SSCs cultured in a serum-free medium containing 10 ng/mL GDNF, 75 ng/mL GFRα − 1, and 1 ng/mL bFGF in the presence of 1 µg/mL 5H-purin-6-amine. The cells were examined using bright field and fluorescence microscopy. (**B**) Germ cells cultured in a serum-free medium with 5H-purin-6-amine were immunostained with the markers for undifferentiated spermatogonia (PLZF and GFRα1), germ cell lineage (VASA), and differentiating germ cells (c-Kit), and isotype IgG negative control (Alexa Fluor 568-conjugated goat anti-mouse, Alexa Fluor 568-conjugated donkey anti-rabbit, and Alexa Fluor 568-conjugated donkey anti-goat). (**C**) Effects of 1 µg/mL Bu and 1 µg/mL 5H-purin-6-amine on *Lhx1* (undifferentiated spermatogonia or spermatogonial stem cell marker) and *Pgk2* (from meiotic spermatocytes to post meiotic spermatid marker) expression were evaluated using real-time PCR analysis. Bu, *n*-butanol fraction. Values are mean  ±  SEM (n = 3 established independent cultures for each treatment). A statistically significant difference (*P* < 0.05) is displayed as different letters at the top of the column. Scale bars: (**A**,**B**) = 100 µm.

Germ cells cultured with 5H-purin-6-amine formed normal-shaped germ cells enriched for SSCs clumps (Fig. 4A). Furthermore, the clump size in the 1 μg/mL 5H-purin-6-amine treatment group was larger than the control after 1 week of culture (Fig. 4B). To identify and evaluate the characteristics of germ cells enriched for SSCs, PLZF and GFRα1 (GDNF family receptor alpha 1) were used as undifferentiated spermatogonia markers. VASA, also known as DDX4 (DEAD box polypeptide 4), was used as an essential marker to verify the characteristics of germ cell lineage. Conversely, c-Kit (KIT proto-oncogene receptor tyrosine kinase), known as a germ cell differentiation marker, was used as a negative marker for ability to self-renewal of SSCs in culture. Normal expression of PLZF, GFRα1, and VASA were observed in germ cells cultured with the 1 μg/mL 5H-purin-6-amine. Additionally, no expression of c-Kit means that cultured germ cells were not differentiated (Fig. 4B). Thus, our results suggested that the 5H-purin-6-amine could effectively enhance germ cell proliferation including SSCs.

### Expression of *Lhx1* and *Pgk2* in Cultured Germ Cells with *n*-butanol Fraction or 5H-purin-6-amine

To investigate LIM homeobox 1 (*Lhx1*) and phosphoglycerate kinase-2 (*Pgk2*) mRNA expressions, germ cells were cultured for 1 week with Bu or 5H-purin-6-amine 1 µg/mL and then analyzed by using qRT-PCR. The *Lhx1*, which is mainly expressed in undifferentiated spermatogonia, was expressed at relatively greater levels in germ cells cultured with 1 µg/mL Bu (2.7 ± 0.6 fold) and 1 μg/mL 5H-purin-6-amine (3.9 ± 1.7 fold) compared to control, although this was not statistically significant (Fig. 4C). Conversely, *Pgk2*, the marker for male germ cells in the meiotic and post-meiotic stages, was expressed at significantly lower levels than control, indicating that 1 μg/mL Bu (0.05 ± 0.02 fold) and 1 μg/mL 5H-purin-6-amine (0.0093 ± 0.0068 fold) decreased the ability of differentiation (Fig. 4C). Therefore, these results showed that 1 μg/mL Bu or 1 μg/mL 5H-purin-6-amine allowed germ cells enriched for SSCs to maintain their self-renewal and decrease differentiation *in vitro*.

### Functional Activity of SSCs Cultured with 5H-purin-6-amine *In Vivo*

**Figure 5:**
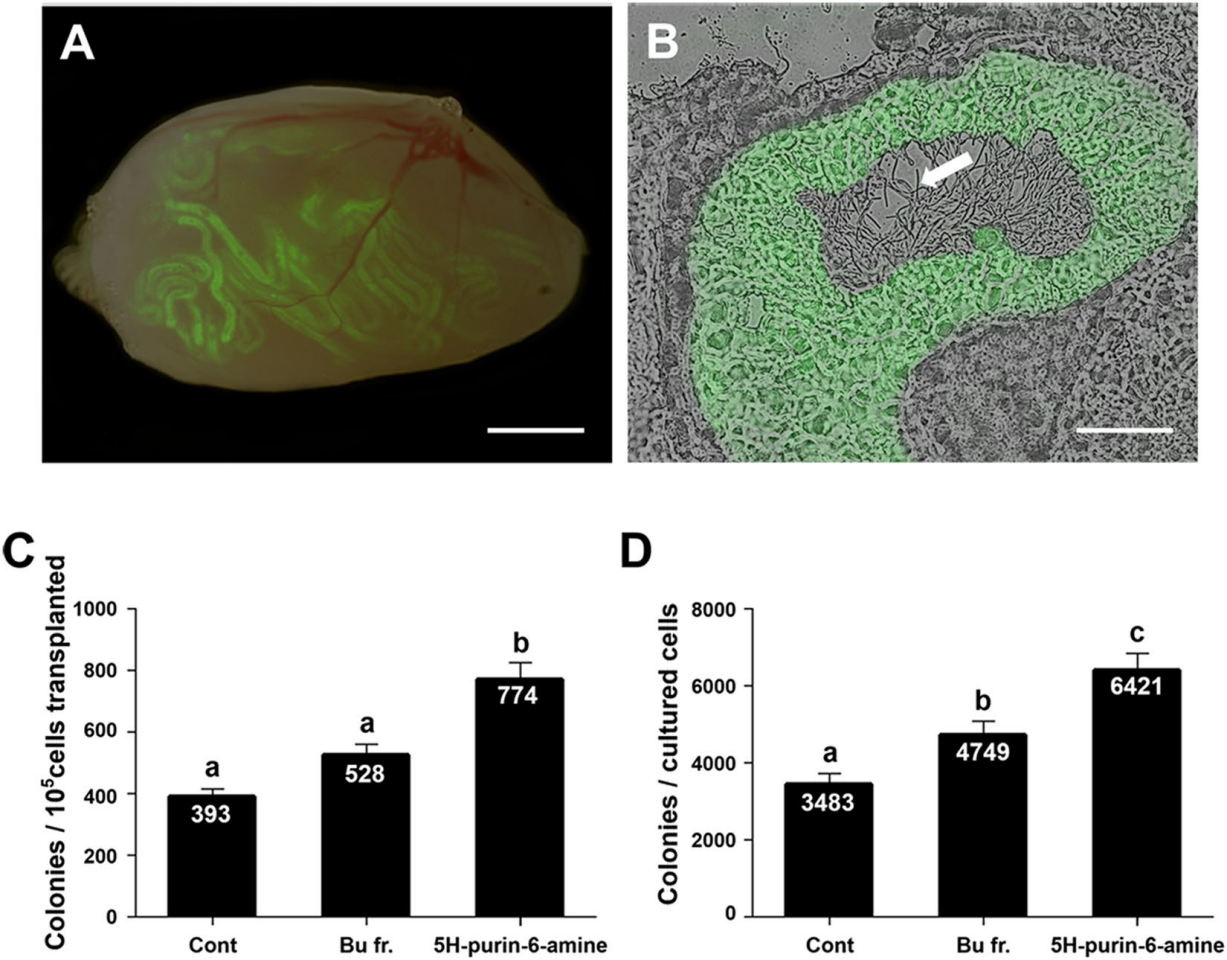
Analysis of functional activity of SSCs cultured with *S. sarmentosum* through germ cell transplantation. (**A**) The dark-field fluorescence image in a recipient testis after transplantation of germ cells cultured with 1 μg/mL 5H-purin-6-amine isolated from *S. sarmentosum*. In the recipient seminiferous tubules, the colonies derived from donor SSCs were distinctly green. (**B**) Cryosection of recipient testis showed GFP expressing colonies derived from donor SSCs, indicating complete spermatogenesis in the lumen of seminiferous tubules. Complete spermatogenesis is evidenced by the presence of sperm (white arrow). (**C**) The number of colonies per 10^5^ cells transplanted with 1 µg/mL Bu or 1 µg/mL 5H-purin-6-amine. (**D**) The relative number of SSCs recovered after culture with 1 µg/mL Bu or 1 µg/mL 5H-purin-6-amine. The total number of mice/testes used for testis analysis was 8/13, 9/13, and 8/13 in the control (cultured only with GDNF at 10 ng/mL), Bu (cultured with GDNF at 10 ng/mL and 1 µg/mL *n*-butanol fraction), and 5H-purin-6-amine (cultured with GDNF at 10 ng/mL and 1 µg/mL 5H-purin-6-amine), respectively. Bu, *n*-butanol fraction. Scale bars: (**A**) = 2 mm; (**B**) = 50 µm.

The largest increase in proliferation rate of germ cells cultured with 1 μg/mL 5H-purin-6-amine does not indicate the stem cell activity of SSCs. Therefore, a germ cell transplantation was performed to estimate the effects of the 5H-purin-6-amine on SSC functions because it is only the most accurate method available to quantify the number of SSCs^30, 31^. Germ cells cultured with 1 μg/mL Bu and 1 μg/mL 5H-purin-6-amine were used for transplantation into recipient mice. Two months after transplantation, colonies longer than 1 mm in length derived from donor SSCs were counted to quantify the stem cell content (Fig. 5A). Because the donor cells expressed GFP, donor-derived colonies could be counted using a fluorescence microscope. In addition, we confirmed that SSCs from donor GFP cells were differentiated into sperm through spermatogenesis (Fig. 5B). The number of colonies per 10^5^ transplanted germ cells was significantly different between the control and 1 μg/mL 5H-purin-6-amine (Control: 393.7 ± 26.0 colonies, 5H-purin-6-amine: 774.3 ± 51.4 colonies; *P* < 0.05; Fig. 5C). Moreover, the colonies per cultured cells were normalized to the number of recovered cells after 1 week of culture, and thus 1 μg/mL 5H-purin-6-amine showed a significant difference (6421.0 ± 431.8 colonies) compared to the control (3483.8 ± 258.3 colonies) as well as 1 μg/mL Bu (4749.3 ± 353.7 colonies) (Fig. 5D). Hence, the results showed both treatment groups increased the activity of SSCs, especially 1 μg/mL 5H-purin-6-amine, indicating that the Bu and 5H-purin-6-amine isolated from *S. sarmentosum* could improve real SSC populations within germ cells.

## Discussion

In this study, we demonstrated that an enhanced SSC proliferation rate may be attributed to the 5H-purin-6-amine of *S. sarmentosum* at 1 µg/mL concentration. Moreover, the result was supported by our overall data which were collected from the proliferation rate and the functional assays, such as gene and protein expression analyses and transplantation.

The fractionation process is usually performed to obtain organic materials with polarity in order of He < MC < EA < Bu. The significant difference in the *S. sarmentosum* extract could be interpreted as follows: regarding the positive effect of all fractions, the complex interactions between each fraction might cause the germ cells enriched for SSCs proliferation rate to increase. Especially, He and EA fractions that have non-polarity, including phenolics, organic acids, and partial fatty acids, which are known as effective natural antioxidants. The mechanism associated with the antioxidant is that phenolics, including –OH or –COOH, with the conjugated aromatic ring suppress lipid oxidation through a reducing process^32–35^. In other words, the high total phenolic level is attributed to high free radical scavenging activity. In spite of good antioxidant effects of all fractions, Bu showed highest proliferation rate compared to others. The ingredients dissolved in Bu mainly correspond with water soluble sugars, amino acids, peptides, and proteins, which can also contribute to antioxidant effects. In the previous studies, the majority of sugars and carbohydrates may promote acidification, whereas amino acids, peptides, or proteins prevent this process^36–38^. Taken together, we speculate that Bu of *S. sarmentosum* mainly contains nitrogenous compounds rather than sugars and carbohydrates. Thus, the Bu was isolated and purified for further analysis as it showed the best effect on proliferation rate among all treatment groups.

*Lhx1* is a protein expressed in undifferentiated spermatogonia and plays an essential role in maintaining the undifferentiated state^39^. Conversely, *Pgk2* is a specific isozyme of *Pgk1* in the testes^40^, indicating that cells expressing *Pgk2* are equal to the male germ cells in the meiotic and post-meiotic stages. The germ cells having stemness property undergo differentiation and subsequently into sperm. Therefore, in this study, greater mRNA levels of *Lhx1* and lower levels of *Pgk2* indicated that the undifferentiated germ cells cultured with the 5H-purin-6-amine had the ability to self-renew, enabling an assessment of the status of undifferentiated spermatogonia.

Immunofluorescence analysis is a useful tool for detection of protein expression at different stages of germ cells enriched for SSCs. We selected undifferentiated and differentiated spermatogonia related marker proteins named PLZF, GFRα1, VASA and c-Kit. In testis, PLZF, expressing only in undifferentiated spermatogonia plays an important role as a spermatogonial specific transcription factor, thereby regulating self-renewal and maintaining the stem cell pool^41^. GFRα1 can also regulate self-renewal and is expressed in undifferentiated spermatogonia^42, 43^ whereas VASA, an RNA helicase, is expressed in germ cells^44^. Finally, c-Kit, which is a tyrosine kinase receptor, acts as a differentiated germ cell marker in adults^45^. This experiment showed the cells expressing PLZF and GFRα1 were similar in number, which indicates that cultured germ cells maintain their characteristics of undifferentiated state (Fig. 4B). In addition, the expression of VASA indicated that cells cultured with the 5H-purin-6-amine retained germ cell properties. There was no observed c-Kit expression, indicated that the proliferation of cultured cells with 5H-purin-6-amine occurred due to self-renewal, not differentiation. Overall, the results suggested that the germ cells enriched for SSCs cultured with 5H-purin-6-amine maintained their undifferentiated state.

Our study investigated real components of natural plant extracts that improved germ cells enriched for SSCs self-renewal ability in mice. Therefore, transplantation was conducted to directly quantify stem cell functional activity. In particular, these results demonstrated that addition of 5H-purin-6-amine of *S. sarmentosum* to *in vitro* culture contributed improvement of SSC self-renewal, and the content of true SSCs was verified by transplantation. Here, we observed an approximate two-fold increase in the number of colonies compared to the DMSO control after transplantation of germ cells cultured with 5H-purin-6-amine. These findings strongly support the finding that 5H-purin-6-amine helps to increase the ratio of real SSC populations, indicating that the compound could be related to the regulation of SSC self-renewal with retaining its stemness.

Interestingly, we found that recovery rate of germ cells cultured with Bu 1 μg/mL seems to be higher than 5H-purin-6-amine 1 μg/mL (Fig. 2 and 3B). In contrast to results from *in vitro* proliferation rate, the concentration and total number of SSC cultured with 5H-purin-6-amine 1 μg/mL was higher than Bu 1 μg/mL as shown in Fig. 5C and D. These results could be interpreted as indicating that *in vitro* cultured germ cells do not a pure population of SSCs but more likely a mixture of spermatogonia and differentiating germ cells. Thus, it is well known that true SSC can be unequivocally identified by functional transplantation assay, representing the clonal expansion of an individual stem cell^30, 31^. Therefore, this finding indicated that 5H-purin-6-amine 1 μg/mL would be main molecule for increase in the number of true SSCs although recovery rate of germ cells was lower than Bu 1 μg/mL.

Importantly, there was a significant difference regarding germ cells enriched for SSCs proliferation with *S. sarmentosum* extract, which exhibited pharmacological action unlike other natural plant extracts. Especially, 1 μg/mL Bu showed higher germ cells enriched for SSCs proliferation than other because it may play an essential role as a reactive oxygen species (ROS) inhibitor^46, 47^. ROS inevitably occur in metabolically active testes, which could adversely affect the testes tissue or cells^48^. Therefore, the ROS inhibitor is essential for maintaining healthy testis metabolism. Furthermore, the *S. sarmentosum* extract is an effective antioxidant, as shown in other studies, although we did not measure the ROS level in the *S. sarmentosum* extract^46^.

*S. sarmentosum* extract is known that it could suppress the proliferation of various cancer cells^28^. Interestingly, Bai *et al*.^49^ reported that inhibitory effect of *S*. sarmentosum on cancer cell proliferation was through the down-regulation of Hedgehog (Hh) signaling pathway. Hh pathway normally regulates embryonic development and tissue repair, increasing the expression of Nanog in consort with the loss of oncosuppressor p53^50^. Moreover, it is also reported that Hh pathway have effects on promotion of stemness/self-renewal in stem cells, especially germline stem cells^51, 52^. However, it can also develop tumor in hyperactive case^53^. Thus, we speculated that optimal gene expression of Hh pathway is required for SSCs proliferation, which is likely to be controlled by *S. sarmentosum* extract. In particular, it shown that SSC proliferation was resulted from culture with 5H-purin-6-amine in this study, which is might due to regulation of Hh pathway by 5H-purin-6-amine. However, further studies will be warranted to investigate the molecular targets or pathways of 5H-purin-6-amine because SSC proliferation is associated with complex signaling pathway such as PI3K/AKR, SFK^43^.

In conclusion, to the best of our knowledge, this is the first report to investigate the effects of 5H-purin-6-amine on SSC self-renewal. Moreover, the compound allows to obtain SSCs in large quantities along with their stem cell properties. These factors ultimately would provide the opportunity to develop treatments for male infertility. In brief, we demonstrated the capacity of 5H-purin-6-amine to maintain the status of self-renewal in SSCs, which highlights the potentially promising role of effective natural plant extracts in stem cell therapies. Our findings also contribute the 5H-purin-6-amine isolated from *S. sarmentosum* to a database for drugs and medicinal use.

## Materials and Methods

### Extraction and Fractionation of Natural Plants

Total 11 natural plants (*Morus bombycis, Lycium chinense, Cuscuta chinensis, Morinda officinalis, Chrysanthemum zawadskii, Torreya nucifera, Portulaca oleracea, Equisetum hyemale, Crataegus pinnatifida, Dioscorea batatas, Sedum sarmentosum*) were used in this study. Leaves of these plants were cultivated and collected from Anseong, Korea. Fresh leaves were dried for 3 days and ground, powdered leaves were extracted using ethanol (EtOH; 8 L × 10) under reflux at 65–75 °C. The filtration was concentrated until dry *in vacuo* to form a dark-green material of EtOH extract. To extract *S. sarmentosum* (*S. sarmentosum*), the EtOH extract was suspended in H_2_O, and then successively partitioned using *n*-hexane, methylene chloride (CH_2_Cl_2_), ethyl acetate (EtOAc), and *n*-butanol (BuOH).

### Isolation of *Sedum sarmentosum* Compounds from the *n*-butanol Fraction

This experiment was conducted by previous reported paper related to isolation of *S. Sarmentosum* compounds^54^. A portion of the *n*-butanol fraction (Bu) was subjected to medium pressure liquid chromatography (MPLC) on silica gel eluted with a gradient of CHCl3-MeOH (100:0 → 50:50) to obtain 10 fractions. Fraction 2 was recrystallized under CHCl3-MeOH to give Bu 2. Fraction 6 was repetitively chromatographed on a Sephadex LH-20 column to obtain 7 fractions (6-1 to 6-7) and fraction 6-3 was recrystallized to give Bu 6-3. Fractions 8 and 9 were repetitively chromatographed on MPLC eluted with CHCl3-MeOH (80:20 → 50:50) to obtain 5 (8-1 to 8-5) and 6 (9-1 to 9-6) fractions, respectively. Fraction 8-3 was separated using a Sephadex LH-20 column to obtain 7 fractions (8-3-1 to 8-3-7), including Bu 8-3-3. Fractions 9-4 and 9-5 were separated using a Sephadex LH-20 column to obtain 5 (9-4-1 to 9-4-5) and 7 (9-5-1 to 9-5-7) fractions, respectively, including Bu 9-4-5 and Bu 9-5-5.

### Experimental Animals

Animal care and all animal experiments were approved by the Animal Care and Use Committee of Chung-Ang University (IACUC Number: 2015-00016). The experiments were performed in accordance with the *Guide for the Care and Use of Laboratory Animals* published by National Institutes of Health. Every mice were maintained under semi-barrier conditions at 21 ± 2 °C, 55 ± 10% humidity, and a 12:12 light:dark cycle. Especially, busulfan treated or transplanted mice were maintained by using filter-topped cages, resulting in protection of the mice from environmental contamination. This study used animals of the C57BL/6-TG-EGFP (designated C57-GFP; Jackson Laboratory) strain, which expresses the green fluorescent protein (EGFP) gene in all cells, thereby allowing the donor cells to be easily recognized in non-EGFP recipient mice. The recipient C57BL/6 mice were obtained from Harlan Laboratories (Indianapolis, IN, USA).

### Isolation and *In Vitro* Culture of Germ Cells Enriched for Spermatogonial Stem Cell

Unless otherwise stated, all reagents were purchased from Sigma-Aldrich. Testis cells, including SSCs, were isolated from 6- to 8-day-old C57-GFP mice. The cell population enriched for SSCs was isolated as previously described, with minor modifications^55^. After collecting fresh testes, the seminiferous tubules were decapsulated and washed in Dulbecco’s phosphate buffered saline (DPBS; Life Technologies, Grand Island, NY, USA). To obtain single cells, the seminiferous tubules were incubated in a 2:1 solution of 0.25% trypsin-EDTA (Invitrogen) and 7 mg/mL DNase I (Roche, Basel, Switzerland) in DPBS at 37 °C for 5 min. The enzyme was inactivated by supplementing with 10% fetal bovine serum at a final concentration (FBS; GE Healthcare Life Sciences, Pittsburgh, PA, USA). To remove large pieces of undigested tunica or tubules after digestion, the cell suspension was filtered through a 40-µm pore size nylon mesh (BD Biosciences, San Jose, CA, USA). Cell viability was determined using trypan blue exclusion, and testis cells that had a viability more than 95% were used for further experiments. The single-cell suspension was centrifuged at 600 × *g* for 7 min at 4 °C, and then 5 × 10^6^ cells/mL were resuspended in Dulbecco’s modified Eagle medium (DMEM; Life Technologies) containing 10% FBS, 2 mM l-glutamine, 0.1 mM β-mercaptoethanol, 100 U/mL penicillin, and 100 µg/mL streptomycin. Testis cells (10 × 10^6^) were overlaid on 2 mL of 30% Percoll solution and centrifuged to separate erythrocytes and debris from the cell suspension. The pellet was resuspended and purified for germ cells enriched for SSCs by magnetic-activated cell sorting (MACS) using anti-Thy-1 antibody microbeads (Miltenyi Biotech, Auburn, CA, USA) as previously described^56^. After purification, Thy-1-positive (Thy-1^+^) cells were placed on 12-well culture plates containing mitotically inactivated SIM mouse embryo-derived thioguanine- and ouabain-resistant (STO) feeder cells. The cells were cultured in mouse serum-free medium (mSFM) including 10 ng/mL glial cell line-derived neurotrophic factors (GDNF; R&D Systems, Minneapolis, MN, USA), 75 ng/mL GDNF family receptor α1 (GFRα1; R&D Systems), and 1 ng/mL basic fibroblast growth factor 2 (bFGF2; BD Biosciences) as previously reported^57–59^. The culture medium was replaced every 2–3 days, and the cultured cells were passaged in a 1:2 or 1:3 ratio every week.

### Germ Cell Proliferation

To assess the cell proliferation rate, the germ cells (8–17 passages) including SSCs were plated on a 24-well plate with serum free culture medium at a density of 2 × 10^5^ cells/well and cultured for 1 week. The culture medium contained 10 ng/mL GDNF as a self-renewal promoting factor and *S. sarmentosum* extract or fractions dissolved in dimethyl sulfoxide (DMSO) at concentrations of 0.1, 1, or 10 µg/mL. Control cells were treated with DMSO along with 10 ng/mL GDNF. After 1 week, the cultured germ cells were collected and GFP-positive cells were counted. After counting, the difference in the proliferation rate was normalized comparing the control and treatment groups using the following equation (1):

1 $$\text{Normalized}\,\text{data}\,( \% )=\frac{\text{Number}\,\text{of}\,\text{recovered}\,\text{cells}\,\text{after}\,\text{culture}}{\text{Number}\,\text{of}\,\text{recovered}\,\text{cells}\,\text{after}\,\text{culture}\,\text{in}\,\text{the}\,\text{control}}\times 100$$

The formula for normalization was on the basis of previous reported formula to understand the data more easily. The equation is calculated on the assumption that the number of germ cells was increased than initial number in control. Actually, the number of germ cells enriched for SSCs was increased 1.98-fold (2 × 10^5^ to 3.8 × 10^5^) on the average^59^.

### Immunocytochemistry

After 1 week of culture, the germ cells were fixed for 30 min at 37 °C with 4% paraformaldehyde and permeabilized for 10 min at room temperature in 0.1% Triton X-100 with DPBS. For blocking, the cells were incubated with 5% (w/v) bovine serum albumin (BSA) for 1 h, and then with a mouse anti-human promyelocytic leukemia zinc finger (PLZF, 1:200; EDM Millipore, Billerica, MA, USA), rabbit anti-human glial-derived neurotrophic factor family receptor alpha 1 (GFRα1, 1:200; Abcam, Cambridge, UK), rabbit anti-human DDX4 (VASA, 1:200; Abcam), and goat anti-mouse c-Kit (1:200; Santa Cruz Biotechnology, Dallas, Texas, USA), and rabbit anti-Ki67 (1:200; Abcam) primary antibody at 4 °C for 12 h. After washing 3 times with DPBS, the cells were incubated with an Alexa Fluor 568-conjugated goat anti-mouse IgG, Alexa Fluor 568-conjugated donkey anti-rabbit IgG, and Alexa Fluor 568-conjugated donkey anti-goat IgG at room temperature for 1 hr. After incubation with the secondary antibody, the cells were washed with DPBS. VectaShield mounting media including DAPI was used to mount the cells. The labeled cells were analyzed using a Nikon TS-1000 microscope with NIS Elements imaging software (Nikon, Chiyoda-ku, Tokyo, Japan).

### Quantitative Real-time Polymerase Chain Reaction

Total RNA was purified and qRT-PCR was conducted using SYBR Green PCR master mix in accordance with the manufacturer instructions. The primers used for the analysis are shown in Table 1. The mRNA expression was normalized based on the level of glyceraldehyde-3-phosphate dehydrogenase (GAPDH) mRNA. The data are shown as mean ± SEM, which was determined from three independent experiments performed in triplicate.

**Table 1:** Primers used for qRT-PCR.

Markers	Gene	Forward primer	Reverse primer
Undifferentiated spermatogonia	*Lhx1*	CCCAGCTTTCCCGAATCCT	GCGGGACGTAAATAAATAAAATGG
Differentiated spermatogonia	*Pgk2*	TGCCATCCCAAGTATCAA	TCAGCAACAGGCTCTAAT

### Germ Cells Transplantation

Prior to C57-GFP donor cell transplantation, the recipient C57 mice were treated with 45 mg/kg body weight busulfan at 6 weeks of age to remove the endogenous germ cells. The germ cells (8–11 passages) were recovered after 7 days culture and concentrated to a density of 1.0 × 10^6^ cells/mL. Avertin and medetomidine diluted 1:1 was used to anesthetize the recipient mice. The donor cells were transplanted through the efferent ducts into the recipient mice testes, as previously described^30, 31^. Approximately 8 µL of the donor cell suspension was injected into the recipient testes, filling 80% of the seminiferous tubules.

### Spermatogonial Stem Cell Activity Analysis

Two months after transplantation, the recipient C57 mice were euthanized to analyze the testes. The collected testes were decapsulated. The number of fluorescent donor colonies greater than 1 mm in length was counted using fluorescence microscopy to quantify the SSCs, as previously described^60^. The number of colonies was calculated as the number of colonies per 10^5^ cells transplanted using the following equation (2):

2 $${\rm{Colonies}}/{10}^{5}\,\text{cells}\,\text{transplanted}=\frac{{\rm{Number}}\,{\rm{of}}\,{\rm{colonies}}\times {10}^{5}}{{\rm{Number}}\,{\rm{of}}\,{\rm{cells}}\,{\rm{Transplanted}}}$$

The total number of colonies was expressed as the number of colonies per total number of cells recovered after culture to indicate the potential effects of the compound of *S. sarmentosum* on SSC proliferation, using the following equation (3):

3 $${\rm{Colonies}}/\text{total}\,\text{number}\,\text{of}\,\text{SSCs}\,\text{recovered}=\frac{\text{Number}\,\,{\rm{of}}\,{\rm{colonies}}\,\times \,{\rm{Total}}\,{\rm{number}}\,{\rm{of}}\,{\rm{cells}}\,{\rm{cultured}}}{{\rm{Number}}\,{\rm{of}}\,{\rm{cells}}\,{\rm{transplanted}}}$$

Three months after transplantation, the testes were collected, cryosectioned, and histologically visualized using fluorescence microscopy to determine the level of donor spermatogenesis in recipient testes.

### Statistical Analysis

Statistical analysis of all data was determined using SPSS software (version 21; IBM Inc., New York, USA) and Prism software (version 5.03; GraphPad, La Jolla, CA, USA). The data were analyzed using one-way analysis of variance (ANOVA) followed by Tukey’s honestly significant difference (HSD) test, and the tests were one-tailed. The data are expressed as mean ± SEM, and the level of significance was determined by *P* < 0.05. Unless otherwise stated, all experiments were performed in triplicate.
